# The HGF/SF Mouse Model of UV-Induced Melanoma as an In Vivo Sensor for Metastasis-Regulating Gene

**DOI:** 10.3390/ijms18081647

**Published:** 2017-07-28

**Authors:** M. Kathryn Leonard, Nidhi Pamidimukkala, Gemma S. Puts, Devin E. Snyder, Andrzej T. Slominski, David M. Kaetzel

**Affiliations:** 1 Department of Biochemistry and Molecular Biology, School of Medicine, University of Maryland-Baltimore, Baltimore, MD 21201, USA; KLeonard@som.umaryland.edu (M.K.L.); NPamidi@umaryland.edu (N.P.); GCook@som.umaryland.edu (G.S.P.); Devin.Sharp@som.umaryland.edu (D.E.S.); 2 University of Maryland Greenebaum Comprehensive Cancer Center, University of Maryland-Baltimore, Baltimore, MD 21201, USA; 3 Department of Dermatology, Comprehensive Cancer Center, University of Alabama at Birmingham, Birmingham, AL 35249, USA; aslominski@uabmc.edu; 4 Cancer Chemoprevention Program, and Nutrition Obesity Research Center, University of Alabama at Birmingham, Birmingham, AL 35249, USA; 5 Pathology and Laboratory Medicine Service, VA Medical Center, Birmingham, AL 35249, USA

**Keywords:** HGF, melanoma, metastasis, genetically engineered mouse models

## Abstract

Cutaneous malignant melanoma is an aggressive and potentially lethal form of skin cancer, particularly in its advanced and therapy-resistant stages, and the need for novel therapeutics and prognostic tools is acute. Incidence of melanoma has steadily increased over the past few decades, with exposure to the genome-damaging effects of ultraviolet radiation (UVR) well-recognized as a primary cause. A number of genetically-engineered mouse models (GEMMs) have been created that exhibit high incidence of spontaneous and induced forms of melanoma, and a select subset recapitulates its progression to aggressive and metastatic forms. These GEMMs hold considerable promise for providing insights into advanced stages of melanoma, such as potential therapeutic targets and prognostic markers, and as in vivo systems for testing of novel therapies. In this review, we summarize how the HGF/SF transgenic mouse has been used to reveal metastasis-regulating activity of four different genes (*CDK4^R24C^*, *survivin* and *NME1/NME2*) in the context of UV-induced melanoma. We also discuss how these models can potentially yield new strategies for clinical management of melanoma in its most aggressive forms.

## 1. Introduction

Melanoma is the most lethal variant of skin cancer, with melanoma incidence and mortality rising dramatically over the past 20 years in both men and women [1]. Exposure to ultraviolet radiation (UVR) is now widely accepted as a major cause of malignant cutaneous melanoma and skin cancer-related deaths [2]. While surgical resection of skin-localized melanomas in their early stages (radial growth phase) is frequently curative, invasive melanomas at vertical growth phase often progress to metastatic disease and death. Despite the advent of inhibitors of constitutively-active BRAF (e.g., vemurafenib) and immune checkpoints (e.g., antibodies against programmed cell death protein 1 (PD-1), programmed death-ligand 1 (PD-L1) or cytotoxic T-lymphocyte-associated protein 4 (CTLA-4), current therapies rarely provide durable responses in advanced melanoma. Moreover, clinical management of melanoma patients is impaired by a lack of molecular markers that reliably identify lesions with elevated risk for metastasis and/or disease progression.

Genes that regulate the metastatic process in melanoma provide unique tools for discovery of new therapies and prognostic markers. To date, candidate metastasis-regulating genes (MRGs) have been identified in studies using established melanoma cell lines, with a spectrum of cell culture and mouse xenograft models used to assess their impacts on growth and metastatic phenotypes. While melanoma cell lines have proven invaluable models for discovery, they are imperfect representations of the melanoma tumors from which they were isolated. Most were selected for proliferation on artificial two-dimensional plastic surfaces, maintained under a continual mitotic stimulus of fetal bovine serum, and subjected to extensive passaging that frequently leads to alterations in genotype and phenotype. Genetically-engineered mouse models (GEMMs) of cancer represent a powerful in vivo approach for measuring metastasis-regulating activity of candidate MRGs in the context of an intact tumor microenvironment and immune system. A myriad of GEMMs have been developed for study of melanoma [3], some of which can indeed progress to metastatic disease and are discussed in articles within this compendium. This review is focused specifically on the hepatocyte growth factor/scatter factor (HGF/SF) mouse model of UV-induced melanoma as a means for measuring metastasis-regulating activity of genes in vivo. Such applications of the HGF/SF model hold promise for elucidating molecular mechanisms underlying the metastatic process, and for the development of new therapeutic and prognostic tools to manage the disease more effectively.

## 2. Application of the Hepatocyte Growth Factor/Scatter Factor (HGF/SF) Transgenic Mouse Model as a Sensor of Metastasis-Regulating Genes (MRG) Activity: Working Principles

**Figure 1:**
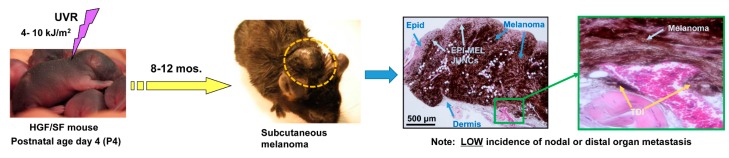
Ultraviolet radiation-induced melanoma in the hepatocyte HGF/SF transgenic mouse (Jarrett et al., 2013) [15]. EPI-MEL JUNCs, epithelial-melanoma junctions; TDI, transdermal invasion with invasion of subcutis.

The HGF/SF strain of transgenic mice overexpresses HGF/SF in tissues under control of the metallothionein (MT) gene promoter [4]. HGF/SF mice also exhibit spontaneous neoplasms of both mesenchymal and epithelial cell origins, a profile likely determined by MT promoter activity across diverse cell types. Importantly, HGF/SF mice display a high incidence of subcutaneous melanoma when subjected shortly after birth (postnatal day 4, P4; Figure 1) to a single erythemal dose of ultraviolet radiation (UVR; 4–8 kJ/M^2^; 35% UV-A/65% UV-B/0.3% UV-C) [5]. The sensitivity of HGF/SF mice to UVR-induced melanoma has been attributed in large part to an HGF-induced shift of mouse skin melanocytes from their normal localization within the hair follicle to more external sites (i.e., UV-exposed) within the epidermis and at the dermal–epidermal junction. The externalization of melanocytes in HGF/SF mouse skin resembles that seen in human skin and is rarely observed in other GEMMs of melanoma [4]. Interestingly, the UV-induced melanoma phenotype of the HGF/SF mouse has been demonstrated in both the non-pigmented FVB and highly-pigmented C57BL/6 genetic backgrounds [6].

The K14-SCF mouse model, which expresses stem cell factor (SCF) in epidermal keratinocytes under control of the keratin 14 promoter [7], also exhibits retention of melanocytes in the interfollicular layer of skin yet is melanoma-resistant [8]. This strongly suggests externalization of melanocytes is not sufficient for UV-induced melanomagenesis and that expression of the HGF/SF transgene itself contributes to the melanoma phenotype.

Cutaneous melanoma lesions usually first appear at 6–12 months post-UVR treatment in 60–70% of irradiated HGF/SF mice (Figure 1), and they resemble human melanoma in a number of respects. For example, melanoma cells form junctions with epithelial cells in the epidermal layer of skin [4,9,10], a similarity with human melanomas rarely seen in other GEMM systems (Figure 1). During malignant progression, UV-induced HGF/SF melanomas also frequently exhibit loss or inactivating mutations within regions of the p16^INK4A^*CDKN2A* gene that encode the p16^INK4A^ tumor suppressor protein [11]. Loss of p16^INK4A^ expression and/or function is also exhibited in the progression of many human melanomas [12]. In addition, constitutive expression of HGF/SF results in activation of the receptor tyrosine kinase and proto-oncogene MET, a growth-promoting autocrine loop characteristic of many human melanomas [10]. The oncogenic signaling loop of HGF/SF and MET is also not represented in other GEMM systems as they often utilize the *BRAF^V600E^* mutation, which is only present in 35–50% of human melanomas, to aid in the induction of melanomas. As such, the HGF/SF GEMM may better model the other 50% of human melanomas not expressing mutant *BRAF*.

**Figure 2:**
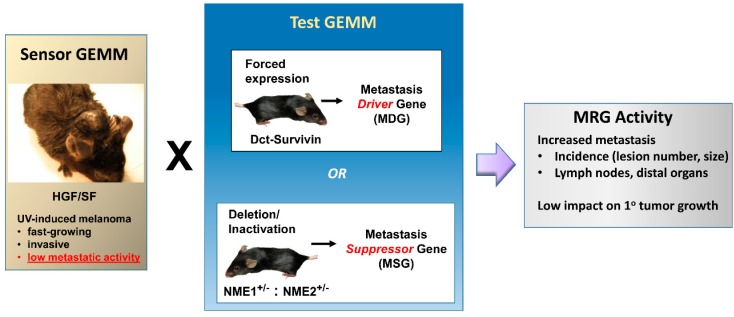
The HGF/SF transgenic mouse as a genetically-engineered mouse model (GEMM) sensor for metastasis-regulating activity (MRG) of genes. Schematic depicts the HGF/SF transgenic mouse as a “*Sensor*” to be bred (X) with “*Test*” GEMMs harboring altered expression of candidate MRGs.

UV-induced melanomas in the HGF/SF model are composed of heavily pigmented epithelioid and dendritic melanocytes with high proliferative potential. Although they readily invade the dermis and adjacent subcutaneous structures (Figure 1), they rarely show evidence of metastasis within four months of their initiation. In this sense, they can be viewed as poised for progression to metastatic activity upon acquisition of gene expression or mutational profiles that drive further progression or the metastatic phenotype itself. This “non-metastatic but poised state” renders them highly suitable for in vivo validation of candidate MRGs (Figure 2). To date, this principle has been exploited to validate MRG activity of four genes: *CDK4^R24C^* [2,13], *survivin* [14], and *NME1/NME2* [15]. Each of these applications of the HGF/SF model as a sensor of MRG activity are reviewed individually in the following section.

## 3. Validation of MRGs Using the HGF/SF Model

### 3.1. Validation of Two Metastasis Driver Genes (MDGs): CDK4^R24C^ and Survivin

#### 3.1.1. CDK4^R24C^

Cyclin-dependent kinases (CDKs) are serine/threonine kinases best known for their cell cycle-regulating functions. CDK4 acts in conjunction with the D-type cyclin proteins to promote the G1-to-S transition, and is opposed in its actions by the CDK inhibitor p16^INK4A^. The *CDK4* gene is often amplified and overexpressed in a variety of cancers including melanoma [16,17,18]. Activating mutations in *CDK4* have also been observed in cancer and are usually directed to the *p16^INK4A^*-binding domain encoded within exon 2 [17]. An example is the arginine_24_-to-cysteine substitution (CDK4^R24C^), which stabilizes the CDK4 molecule by preventing its physical interaction with *p16^INK4A^* [19]. In turn, constitutive activity of CDK4^R24C^ overrides the ability of *p16^INK4A^* to function as a mediator of oncogene-induced senescence [20,21] A transgenic mouse strain engineered on a C57BL/6 genetic background for constitutive expression of CDK4^R24C^ was shown to be prone to two step chemical (7,12-dimethylbenz[a]anthracene (DMBA)/12-*O*-tetradecanoylphorbol-13-acetate (TPA))- and UV-induced melanomas, although the tumors exhibited little metastatic potential [21,22]. Moreover, lifespan of the mice was limited by the incidence of unrelated tumors such as sarcomas and lymphomas. Further evidence of the importance of these pathways is the observation that loss of the *CDKN2A* locus encoding p16^INK4A^ and p14^ARF^, which, respectively, inhibit CDK4/6 and activate the p53 tumor suppressor, are among the most commonly inactivated genes in cancer (including melanoma) [23,24].

To generate a more aggressive mouse model of CDK4^R24C^-dependent melanoma, the CDK4^R24C^ strain was crossed with HGF/SF mice. This hybrid indeed exhibited a more rapid onset of DMBA/TPA- and UVR-induced melanomas than either of the parental strains [25]. Moreover, DMBA/TPA-induced melanomas were more invasive in the hybrid, with hybrid tumors displaying enhanced metastasis to regional lymph nodes and lungs. Organ metastasis was limited to lung tissue, with no lesions detected in liver, spleen, kidney or brain. In a later study, however, liver metastasis was indeed observed in UVB-induced melanomas with the CDK4^R24C^ × HGF/SF model [26]. In effect, these studies represented an early application of the HGF/SF mouse as a sensor for metastasis-regulating activity of a test gene, CDK4^R24C^. A caveat of the study is that both parental strains are prone to induced forms of melanoma, conferring uncertainty as to the relative roles of each transgene in melanoma initiation and growth. Another consideration is that primary melanomas of the hybrid strain grew more rapidly and with more lesions per mouse, suggesting increased cumulative tumor mass could have contributed to the metastatic phenotype in addition to, or in lieu of, a direct impact of CDK4^R24C^ on metastatic potential of the melanoma cell itself. In addition, the germline transmission and constitutive expression of the CDK4^R24C^ transgene across all tissues suggests the possibility that impacts of CDK4^R24C^ on cells in the tumor microenvironment (alone or in combination with HGF/SF) could have contributed to the elevated metastatic activity of melanomas in the hybrid strain. Indeed, paracrine actions of HGF/SF in the HGF/SF model have been shown to impact lung colonization activity of melanoma cell allografts [27]. Overall, however, multiple studies have provided valuable proof-of-principle for the HGF/SF model of UV-induced melanoma as a sensor of MSG activity. To date, the HGF/SF × CDK4^R24C^ hybrid has proven a valuable model of metastatic melanoma for studies of immunological tolerance [22], and impacts of inflammation on resistance to T-cell therapy [28] and metastatic potential [2,25].

#### 3.1.2. Survivin

Survivin is a member of the “Inhibitors of Apoptosis (IAP)” family of proteins, which were first identified in viruses and later in metazoans (reviewed in [29]). Evidence suggests IAP activity of survivin is mediated at least in part via inhibition of caspase activity (primarily caspases 3 and 7) and structural-functional interactions with the protein, nuclear factor kappa-light-chain-enhancer of activated B cells (NF-κB). Survivin expression is associated with fetal tissues, but is not normally seen in adult melanocytes [30] or other differentiated cells [31]. Survivin expression is a hallmark of melanoma [32] and a host of other human cancers, including those of the lung, colon, breast, prostate, and neuroblastoma [33,34,35]. In melanoma, strong positive correlations are seen among survivin expression, metastatic disease, and poor survival [36,37]. Consistent with a cancer-driving function, survivin plays key roles in mitotic control, specifically cytokinesis [38], and chromosomal alignment during mitosis [39]. Using a variety of melanoma cell lines, multiple groups have demonstrated that disruption of survivin expression and/or function confers sensitivity to apoptotic signals, chemosensitization, and inhibits growth as tumor xenografts in mice [40,41]. Survivin has also been shown to confer metastatic activity to the mammary adenocarcinoma cell line R3230AC [42], suggesting a potential metastasis-driving function. Another study has implicated α5β1 integrin as a mediator of survivin-driven metastasis in melanoma cell lines [43].

To investigate potential roles of survivin in the initiation and progression of melanoma in vivo, a transgenic mouse strain was created that provided melanocyte-specific expression of survivin under the transcriptional control of the dopachrome tautomerase (Dct) promoter [14]. Melanocytes isolated from these mice (Dct-survivin) were resistant to UV-induced apoptosis, but their proliferative rate was unaltered. Likewise, formation of DMBA-induced melanocytic nevi was unaffected in the Dct-survivin strain. To assess the impact of survivin in the context of a full melanoma phenotype, Dct-survivin mice were bred with the HGF/SF strain. Melanocytes in the HGF/SF × Dct-survivin hybrid strain exhibited higher resistance via anti-apoptotic mechanisms to UVR treatment in vivo than the parental HGF/SF strain, consistent with the survivin-dependent resistance seen with cultured Dct-survivin melanocytes ex vivo. Survivin overexpression in the hybrid strain also accelerated the onset and density of UV-induced melanomas. Notably, larger melanomas (>5 mm) in the hybrid strain were associated with a significantly higher incidence of lymph node involvement and lung metastasis than seen with similarly-sized melanomas of the HGF/SF strain. The increased metastatic activity of melanomas in hybrid mice was accompanied by more prominent association of melanoma cells with blood and lymphatic vessels (angiotropism), as well as adjacent muscle tissue.

In addition to providing in vivo validation of MDG activity for survivin, the model also yielded in vivo confirmation of earlier observations that survivin confers resistance to apoptosis in melanoma cells. Melanomas that metastasized in the hybrid strain indeed contained significantly fewer apoptotic cells than matched, non-metastatic tumors from HGF/SF mice. Suppression of apoptosis has been suggested as a metastasis-promoting function of cancer cells [44], conferring survival advantage to tumor cells upon acquisition of potential metastasis-driving mutations as well as during dissemination and colonization steps of metastasis. A complicating factor with the model was a significantly higher tumor load in the Dct-survivin × HGF/SF hybrid (two-fold), which could have contributed to the metastasis phenotype. On the other hand, the increase was relatively small, and taken together with minimal effects of survivin on growth rates of melanoma lesions would seem unlikely to account completely for the strong metastasis observed. In addition to revealing MDG activity for survivin in vivo, the Dct-survivin × HGF/SF hybrid strain may be suitable as an in vivo model for testing of survivin-inhibitory therapies in advanced melanoma. Overall, this study provided a further demonstration of the utility of the HGF/SF mouse strain as a sensor for validating the MRG activity of a test gene (survivin) in vivo. In light of the utility of the HGF/SF mouse in revealing metastasis-driving activity of survivin, it could also be of potential application to analysis of other apoptosis-inhibiting proteins such as other IAP factors and members of the Bcl-2 family in a wild-type BRAF background [45]. Nearly all prior studies with anti-apoptotic proteins were conducted in a mutant BRAF background, despite the understanding that HGF-cMET signaling (like that in the HGF/SF model) activates AKT which can lead to suppression of many apoptosis inhibiting genes [45,46].

### 3.2. MSGs: NME1 and NME2

The *NME1* (initially designated *nm23*, for ***N***egative in ***M***etastasis clone ***23***”) gene was identified by virtue of reduced expression of its cognate transcript in a metastatic clone of the mouse melanoma cell line K-1735 [47]. Enforced expression of *NME1* was shown to suppress metastatic potential in a variety of cell lines, and with the notable absence of impacts on proliferative or tumor-forming capacity (reviewed in [48]). An inhibitory activity of *NME1* in cancer metastasis was further suggested by an inverse correlation between *NME1* expression and poor clinical outcome observed across a spectrum of human cancers, including melanoma [49,50]. Together, these findings led to the designation of *NME1* as a metastasis suppressor gene (MSG), a term subsequently extended to all genes sharing the unique ability to suppress metastatic potential of cancer cells without impacting primary tumor growth [51,52,53]. At least 23 MSGs have been described that affect a broad spectrum of cellular processes, and are operative in many different settings of cancer [51]. Due to their selective impact on metastasis, additional study of MSGs holds great potential for elucidating molecular events and processes that specifically regulate the metastatic process. The metastasis-directed actions of MSGs stand in contrast with those of oncogenes and tumor suppressors, whose impacts on tumor growth can obscure their effects on the metastatic process per se.

In addition to *NME1*, nine other homologs (*NME2–NME10*) have been identified [54]. NME homologs 1–4 each harbor a nucleoside diphosphate kinase (NDPK) activity in vitro that catalyzes transfer of γ-phosphate residues between NTPs and NDPs [55] via a phospho-histidine intermediate. Our laboratory also demonstrated NME1 harbors a 3′–5′ exonuclease activity directed to single-stranded DNA substrates [56]. Both enzymatic activities have been suggested to contribute to the metastasis suppressor function of NME1 in melanoma cells [57], although the underlying molecular mechanisms have yet to be identified.

We have shown that NME1 expression promotes repair of UV-induced DNA damage via the nucleotide excision repair pathway in yeast [58] and mammalian cells [59]. Moreover, mice rendered hemizygous-null at the tandemly-arranged *NME1* and *NME2* loci (herein designated *NME1/2^+/−^*) are highly vulnerable to UV-induced melanoma in situ and epidermal inclusion cyst formation on tail skin [59], consistent with a deficit in DNA repair. The DNA repair-promoting activity of NME1 and/or NME2 suggests loss of their expression could promote acquisition of mutations that accelerate melanoma progression. Interestingly, expressions of genes involved in multiple pathways of DNA repair are upregulated in metastatic melanoma [60,61,62,63]. This suggests that viability of metastatic cells may require a return to genomic stability in order for proliferation to be both fast and free of errors, and may indeed contribute to the resistance of metastatic melanoma to therapy [64].

We and others have also shown that NME1 regulates a broad spectrum of RNAs [65,66,67], some of which may mediate its metastasis suppressor function. For example, NME1 regulates expression of numerous RNA species in breast carcinoma cells, with the lysophosphatidic acid (LPA) receptor EDG2 identified as a key target [67]. Both NME1 and NME2 exhibit binding and DNA-cleaving activities upon single-stranded motifs in the *CMYC* [68] and *platelet derived growth factor A* (*PDGFA*) [56,69] promoter regions, and appear to regulate transcription via those interactions [70]. Chromatin immunoprecipitation analyses have confirmed physical association of NME1 with non-B-form elements in the promoter regions of multiple genes, including PDGFA and others [71,72]. Taken together, these studies suggest direct participation of NME1 and NME2 proteins in both DNA repair and gene transcription processes. Such functional duality is not unusual, with a recent report identifying more than 2500 unique pairwise protein associations that encompass the processes of DNA repair and gene transcription [73]. Clearly, both processes could be central to the metastasis suppressor function of NME proteins.

**Figure 3:**
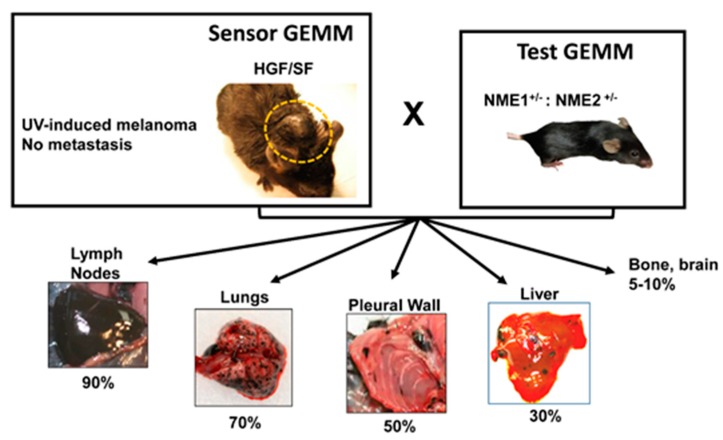
Ablation of the NME1/NME1 locus confers lymph node and organ metastasi to UV-induces melanomas in the HGF/SF transgenic mouse. Schematic depicts the HGF/SF transgenic mouse as a “Sensor GEMM” and the NME1^+/−^;NME2^+/−^ transgenic strain as the “Test GEMM.” Percentages represent percent incidence of mice bearing melanin-pigment metastatic lesions in organs shown. Images have appeared previously in ref. 10 and are used herein with the permission of the journal.

While studies conducted in cancer cell lines strongly suggested NME1 possesses metastasis suppressor activity, the activity had yet to be validated in a model of spontaneous melanoma in vivo. To this end, we crossed the HGF/SF and NME1/2^+/−^ mouse strains in a C57BL/6 genetic background to measure the impact of *NME1/NME2* deficiency on the metastatic potential of UV-induced melanoma [15]. It should be noted that the homozygous-null *NME1/2^−/−^* genotype could not be used in this study, due to death in late gestation or early postnatal ages from severe anemia and other manifestations of impaired erythroid development [74,75]. While UV-induced melanomas of the HGF/SF strain failed to exhibit metastasis to lymph nodes or distal organs, robust metastatic activity was seen in HGF/SF × NME1/2^+/−^ hybrids bearing large (500 mm^3^) melanomas on back skin (Figure 3). Heavily pigmented metastases to draining lymph nodes were observed in 9/10 of these mice, with a majority (7/10) also exhibiting a high number of melanotic metastases in lung. A significant number (3/10) of mice also had substantial metastatic involvement in liver and the thoracic cavity, and to a lesser extent in bone and brain (Figure 3). Metastasis was not observed in melanomas of either strain that arose in other UV-exposed sites, such as the tail, leg/paw or ear. Such melanomas were delayed in their appearance and had much slower growth rates than those arising on back skin. Consistent with the classical definition of an MSG, the *NME1/2^+/−^* genotype did not affect the average growth rate of primary tumors, time of onset of UV-induced melanomas (~180 days), or penetrance of the melanoma phenotype (~70% of irradiated mice). Somewhat unexpectedly, no difference was observed in the invasive index of melanomas between the two strains, suggesting the high metastatic potential conferred by *NME* deficiency was due to impacts on subsequent steps of the metastatic cascade. Cumulatively, these findings confirmed the HGF/SF model as a robust sensor for MRG activity of the test genes *NME1* and *NME2*. Interestingly, a metastatic phenotype was conferred even in the face of one intact copy of *NME1* and *NME2* and retention of a 50% expression level for both proteins, highlighting the sensitivity of the HGF/SF model. Moreover, the HGF/SF model system revealed prototypical MSG activity for *NME1* and/or *NME2* for the first time in an in vivo setting of melanoma, with suppression of metastasis in the absence of an effect on primary tumor growth. Another group has also verified the metastasis suppressor function of *NME1* in the context of a mouse model of carcinogen-induced hepatocellular carcinoma [54].

As with the studies of HGF/SF × CDK4^R24C^ hybrid, our NME1/NME2 deletion was germline in nature and is thus found in all tissues of the NME1/2^+/−^ genotype. This again suggests the possibility that impacts of a test gene (e.g., NME1/2 locus) on metastasis of UV-induced HGF/SF melanomas might be exerted on the tumor microenvironment in addition to, or in the absence of, effects on the melanoma cell itself. While no overt evidence of changes within the microenvironment was apparent in the histological analysis of tumors in our study, the question has yet to be addressed in detail.

Our laboratory is currently exploiting the HGF/SF and NME1/2^+/−^ model system on a number of fronts. The system is now being used to measure the separate impacts of *NME1* and *NME2* on metastatic potential, by crossing individual mouse strains that harbor homozygous-null deletions of either the *NME1* [76] or *NME2* genes [77] with HGF/SF mice. We have also generated a number of stable cell lines from primary and metastatic lesions in HGF/SF and NME1/2^+/−^ mice for genetic manipulation and functional studies. Their C57BL/6 origin will permit allografting in syngeneic C57/BL/6 mice harboring an intact immune system. In light of the DNA repair and transcription functions of *NME1*, we are also using NextGen sequencing to compare mutational and transcriptional profiles in UV-induced melanomas (both primary and metastatic tumors) between the HGF/SF and HGF/SF × NME1/2^+/−^ hybrid strains. These studies will be facilitated by the low frequency of melanoma lesions per mouse (usually one), facilitating comparisons of matched primary and metastatic tumors. It is anticipated that genomic and transcriptomic alterations will be identified that are correlated with the metastatic phenotype and, thus, potentially metastasis-regulating in nature. A clear advantage of this model system for these studies is the genetic homogeneity of the mouse strains under study, with all strains having been incorporated (>6 generations) into C57BL/6 genetic background of our own colony. This provides a considerable advantage over genomic analyses in human melanomas, which are subject to considerable heterogeneity from polymorphisms and mutations in the human population. Candidate metastasis-regulating alterations will be validated to the extent possible by analyzing their impacts on growth and metastatic phenotypes in cell culture systems, as well searching for their potential occurrence in human melanomas. In principle, such alterations could prove valuable as prognostic markers for melanoma progression in human patients.

## 4. Optimization of the HGF/SF Model as a Sensor for MRG Activity

While the HGF/SF model has been used effectively as a sensor for MRG activity, the application can be further optimized in a number of ways. A current impediment to progress is the length of time of a typical experiment, which can take up to a year after the initiating UVR treatment of neonatal mice. We are currently incorporating a melanocyte-specific, homozygous-null of the *p16^INK4Aa^*inactivation of the p16^INK4A^ coding region of the *CDKN2A* gene into the HGF/SF model to accelerate our ongoing studies of MRG activity of *NME* genes. Mice bearing a melanocyte-directed inactivation of the *p16^INK4A^P16INK4A* locus should exhibit a longer lifespan than those bearing a germline deletion, which die prematurely. Introduction of the *p16^INK4A^P16INK4A-*null genotype into HGF/SF mice was shown previously to cut melanoma onset time by more than half, from 6–12 months to 2–3 months [78]. That study also showed that the *p16^INK4A^*-null condition maintained the average multiplicity of UV-induced melanoma lesions to one per mouse, an important consideration for pairwise comparisons between primary and metastatic tumors. Another potential complication was posed in the CDK^R24C^ and *NME* gene studies, in which the genetic alterations were implemented in all cell types/tissues of the mouse. This raised the issue of whether effects on tumor and/or metastatic potential were exerted not only on the melanoma cell itself but also other cells, such as those of the TME. The survivin study [14] revealed how a melanocyte-specific promoter such as the Dct promoter can be used effectively to limit expression of MRG candidates to tumor tissue. The utility of a Dct-driven, doxycycline-inducible transgene encoding a fusion of histone 2B and green fluorescence protein [79,80] illustrates the feasibility of such an approach. In addition, standard Cre-lox approaches to conditional (i.e., melanocyte-specific and time-controlled) deletion should be considered for future analysis of candidate MSGs. Another limitation of the HGF/SF sensor model is that mice must be sacrificed to determine if metastatic lesions are present, unless expensive alternatives such as magnetic resonance imaging (MRI) are available [81,82]. Incorporation of a melanocyte-specific transgene such as a Dct-luciferase module would enable monitoring of primary and metastatic melanoma lesions in living animals with standard imaging equipment.

## 5. Conclusions

With studies in melanoma cell lines providing an increasing list of genes, genetic lesions, and gene expression profiles that exert metastasis-regulating activity, the HGF/SF model of UV-induced melanoma represents a valuable approach for measuring their respective activities in vivo. A primary attribute of the HGF/SF model is the UV-initiated character of its melanomas [83], which recapitulates the UV-dependence of most human melanomas [84]. HGF/SF melanomas also exhibit a number of other characteristics that resemble the human form of the disease, such as the junctional activity observed between melanoma and epithelial cells in skin [4,10], and loss of the tumor suppressor *CDKN2A* during malignant progression [11]. Importantly, melanomas of hybrid crosses of the HGF/SF mouse with three different strains of mice harboring genetic alterations give rise to lymph node and organ metastasis [14,15,25]. In particular, the HGF/SF × NME1/2^+/−^ hybrid exhibits a tropism of metastases (lymph node > lung > liver > bone, brain, etc.) that is nearly identical to that observed in human melanoma [85].

To our knowledge, impacts of the known melanoma drivers *BRAF^V600E^* and *PTEN* inactivation/deletion have not been examined in the context of the HGF/SF mouse model. While the conditional *BRAF^V600E^*/*PTEN* knockout mouse model is well-known for its robust metastatic melanoma phenotype [86], and PI3K/AKT signaling indeed underlies the phenotype [87], these lesions would seem unlikely to provide strong pro-metastatic effects in the HGF/SF mouse where signaling via the RAS/RAF/MAPK and PI3K pathways is already constitutively active [88]. Nevertheless, boosting of signaling pathways initiated by *BRAF^V600E^* expression and *PTEN* inactivation could have unanticipated benefits in accelerating UV-induced melanoma initiation and progression and, thus, may warrant experimental testing.

In addition to providing a system for quantifying MRG activity, HGF/SF hybrids that exhibit robust metastatic melanoma are promising in vivo models for testing of novel therapeutics specifically directed to various aspects of the metastatic process. Of particular interest is the elimination of metastatic cells in both growing and dormant forms, disruption of individual steps in the metastatic cascade, and functional assessment of other relevant molecular targets. As discussed above, the HGF/SF model may not be appropriate for testing of inhibitors of mutant BRAF, the constitutive MET activity and consequent downstream activation of BRAF in HGF/SF melanomas likely precludes the genesis of BRAF-activating mutations. Nevertheless, metastatic variants of the HGF/SF model appear to be a viable option for testing of novel metastasis-directed therapeutics alone and in combination with MAPK inhibitors, immune checkpoint inhibitors, and even conventional therapeutic regimens. As discussed above, detailed comparisons of molecular signatures between non-metastatic melanomas of the HGF/SF strain with metastatic melanomas of HGF/SF hybrid crosses may provide an ideal discovery platform for the diagnostic and prognostic markers that are so acutely needed for management of melanoma patients.

## Abbreviations

CDK	Cyclin-Dependent Kinase
Dct	Dopachrome Tautomerase
GEMM	Genetically-Engineered Mouse model
IAP	Inhibitor of Apoptosis
HGF/SF	Hepatocyte Growth Factor/Scatter Growth Factor
MRGs	Metastasis-Regulating Genes
MDG	Metastasis-Driving Gene
MSG	Metastasis Suppressor Gene
MT	Metallothionine
UVR	Ultraviolet Light Radiation
DMBA	7,12-Dimethylbenz[a]anthracene
TPA	12-O-Tetradecanoylphorbol-13-acetate
NF-κB	Nuclear Factor Kappa-light-chain-enhancer of activated B cells
LPA	Lysophosphatidic Acid
PDGFA	Platelet Derived Growth Factor A
